# Comparison of Two Different Norepinephrine Bolus Doses for Management of Spinal Anesthesia-Induced Maternal Hypotension in Cesarean Section: A Double-Blind Randomized Controlled Study

**DOI:** 10.3390/jcm14248951

**Published:** 2025-12-18

**Authors:** Levent Özdemir, Gönül Sarı Pire, Aslınur Sagün, Mustafa Azizoğlu, Tuğsan Egemen Bilgin

**Affiliations:** 1 Department of Anesthesiology and Intensive Care, Mersin University Faculty of Medicine, Mersin 33110, Türkiye; aslinur_aslan@hotmail.com (A.S.); dryalamaoglu@hotmail.com (M.A.); tugsanb@mersin.edu.tr (T.E.B.); 2 Department of Anesthesiology and Intensive Care, Dr. Behcet Uz Pediatric Diseases and Surgery Training and Research Hospital, İzmir 35210, Türkiye; drgonulsari@gmail.com

**Keywords:** norepinephrine, spinal anesthesia, hypotension, cesarean section

## Abstract

**Background/Objectives**: The bolus dose of norepinephrine (NE) for the treatment of spinal anesthesia-induced maternal hypotension remains unclear. The aim of this study was to compare the efficacy and safety of two different bolus doses of NE in the treatment of spinal anesthesia-induced maternal hypotension during cesarean section (CS). **Methods**: This study has a prospective, randomized and double-blinded design. A total of 150 patients with ASA physical status I and II who underwent CS included the study at a tertiary-care university hospital. Patients were randomly assigned to either the 6 µg NE bolus (Group 6 µg, n = 75) or 8 µg NE bolus (Group 8 µg, n = 75) group. The primary outcome of this study was to determine the success rate of treatment using a 6 or 8 µg NE bolus in cases of maternal hypotension. Secondary outcomes included comparing fetal umbilical blood gas values and APGAR scores in neonates born to mothers who received a NE bolus before delivery, determining the incidence of NE-induced reactive bradycardia and hypertension, and comparing the groups for intraoperative nausea and vomiting. **Results**: The 8 µg group showed fewer hypotensive episodes (245 vs. 174 episodes, *p* = 0.012) and required fewer norepinephrine boluses. Additionally, the success rate of hypotension treatment was higher in the 8 µg group (61.2% vs. 78.5%, *p* < 0.001). There was no difference in APGAR scores between the groups of neonates born to women who received norepinephrine before delivery. However, in the group with 8 µg NE, slightly better results were obtained in blood gas samples, excluding the pH value. Nausea was more frequently observed in the 6 µg group (*p* = 0.046). **Conclusions**: We concluded that an 8 µg bolus administration of NE was more effective than a 6 µg bolus administration in treating maternal hypotension induced by spinal anesthesia, without any increase in the rate of side effects.

## 1. Introduction

Maternal hypotension after spinal anesthesia is a common and serious complication during cesarean delivery [1,2]. The incidence of hypotension during spinal anesthesia for cesarean section (CS) varies in different studies, ranging from 25 to 75% [3]. In these cases, maternal hypotension treatment is usually required using vasopressor boluses. There are also studies that conclude that heart rate (HR) may be a better surrogate than blood pressure when cardiac output is not measured quantitatively in this patient group [4]. In the past, ephedrine was extensively used for this purpose because it preserved uterine blood flow, but it was replaced by phenylephrine due to its negative effects on fetal acid–base balance [5]. Phenylephrine, although long considered the first choice for preventing and treating maternal hypotension, increases vascular resistance due to its pure α-1 agonist effects, can cause bradycardia, and may lead to a decrease in maternal cardiac output [6].

One of the vasopressor agents used in cesarean deliveries recently is norepinephrine. Norepinephrine is the primary catecholamine released by postganglionic adrenergic nerves. It is a potent α-1 adrenergic agonist, with comparatively modest β-agonist activity. It causes marked vasoconstriction with some direct inotropic effects. Norepinephrine (NE) administration results in a higher HR than with comparable doses of phenylephrine [7].

Various infusion doses have been studied to prevent spinal anesthesia-induced maternal hypotension [8,9,10,11]. However, preparing these NE infusions and accessing infusion pumps are not always possible, especially in resource-limited institutions. Therefore, bolus vasopressor administration remains an easy and rapid method. In the literature, a significant portion of studies comparing effective bolus doses of NE are not pure bolus administration (administering bolus in addition to infusion) and have aimed to prevent hypotension rather than treat it [12,13]. Therefore, there are very limited studies investigating the optimal bolus dose of norepinephrine for the treatment of a maternal hypotensive episode, and the optimal dose recommendation is uncertain.

When determining the doses for this study, we chose a 6 µg bolus of NE dose because one study found it to be the effective dose (ED90) for preventing maternal hypotension induced by spinal anesthesia in 90% of patients [14]. In previous studies, ED50-95 values of NE were determined to be between 3.7 and 10 µg. Although our NE dose preferences (6 and 8 µg) may seem close to each other in this study, a recent study found a success rate of 68% for a 7 µg NE bolus dose in treating maternal hypotension, while the success rate for an 8 µg NE bolus was 100% [15]. In this study, the bolus doses of norepinephrine tested are, to our knowledge, the first reported in the literature.

In this study, we evaluated the efficacy, side effects and impact on neonatal outcomes of two different bolus doses of norepinephrine (6 µg and 8 µg) for treating maternal hypotension induced by spinal anesthesia during cesarean delivery.

## 2. Materials and Methods

### 2.1. Study Design and Ethics

A prospective randomized, double-blinded trial conducted in the obstetric theater at Mersin University Hospital from 1 August 2022 to 26 October 2022. The study was approved by the institutional clinical research ethics committee with decision number 2022/560 dated 4 May 2022, and conducted in accordance with the ethical standards of the Helsinki Declaration (2013 review). It was also registered with ClinicalTrials.gov (NCT05502146). The Consolidated Standards of Reporting Trials (CONSORT) system was used for reporting. Preoperative information was provided to the patients included in the study and their written informed consents were obtained.

### 2.2. Patients’ Inclusion and Exclusion Criteria

This study included term singleton pregnancies seeking elective cesarean section, with participants aged 18 to 49 years and classified as American Society of Anesthesiologists (ASA) physical status I–II. Additionally, individuals not using preoperative vasoactive drugs, with intraoperative blood loss not exceeding 750 mL, and with a sensory block not extending above the T4 dermatome level as determined by the pinprick test following spinal block, were included. Participants were excluded if they had any known cardiovascular disease or stage 2 or higher hypertension, defined as a systolic blood pressure (SBP) higher than 140 mmHg or a diastolic blood pressure exceeding 90 mmHg. Participants with known allergies to local anesthetics were excluded, and written informed consent was obtained from all participants.

### 2.3. Randomization and Blinding

A computer-generated sequence was created by the investigators using an online random number generator (randomizer.org). The codes generated for the participants were placed in numbered opaque envelopes. An anesthesiologist, not involved in the patient’s intraoperative management, opened the envelope and prepared the study drug according to the instructions. Multiple syringes were prepared for each patient, each containing a volume of 10 mL but with varying concentrations. A dose of either 6 µg or 8 µg of NE was adjusted in 10 mL syringes and given to a blind anesthetist for administration as a bolus, who then recorded the data. If a participant did not require a norepinephrine bolus, the randomization code was returned to a new similar envelope to be reused with the next mother.

### 2.4. Study Protocol and Measurements

Patients who met the inclusion criteria were randomly assigned to either the 6 µg NE bolus group (Group 6 µg) or the 8 µg NE bolus group (Group 8 µg) for the treatment of spinal anesthesia-induced maternal hypotension. Upon arrival to the operating room, participants were monitored using electrocardiography, pulse oximetry, and non-invasive blood pressure monitoring (GE, Solar™ 8000i, GE HealthCare, Chicago, IL, USA). An 18–20 G venous cannula was inserted, with the first choice being the antecubital region; if this was not possible, the second option was the dorsum of the hand. Lactated Ringer’s solution was administered to the patients at a rate of 15 mL kg^−1^ in the preoperative waiting room, 30 min before the spinal injection. 12.5 milligrams (2.5 mL) bupivacaine (Marcaine^®^ Spinal Heavy 0.5%, Fontenay, France) were injected in the L3–L4 or L4–L5 interspace. The spinal block was performed in the sitting position using a 25 G spinal needle, and the participant was then positioned supine with a left-lateral uterine tilt. Pinprick was used for evaluation of block success 5 min after intrathecal injection. The block was considered successful if the sensory block level was at least at T6.

Baseline blood pressure values were recorded before the spinal block. Systolic blood pressure was then recorded at 2 min intervals for 10 min after the intrathecal injection, and subsequently at 5 min intervals until the end of the surgery. SBP was re-measured 1 min after each norepinephrine (Steradin 4 mg/4 mL, Brussels, Belgium) administration, regardless of routine measurement intervals. Fluid administration continued up to a maximum of 1500 mL. After delivery, an oxytocin bolus (0.5 IU) was delivered over 5 s.

### 2.5. Definitions

The participant was considered to have a hypotensive episode if their SBP was ≤80% of the baseline reading or <90 mmHg from the time of intrathecal injection of local anesthetic until the end of the surgery. A hypotensive episode was managed by injection of an NE bolus, either 6 µg or 8 µg, according to the group code. The hypotensive episode was considered as successfully managed if maternal SBP returned to >80% of the baseline reading and ≥90 mmHg within 6 min. The NE bolus was considered a failure if the maternal SBP did not reach the target value within 6 min after the bolus, or if the SBP dropped below 80% of the baseline reading for two consecutive measurements following the NE bolus. In case of a failed bolus, an additional NE bolus with the same dose was administrated. Patients who had no hypotensive episodes according to established criteria during surgery after spinal anesthesia were not included in group comparisons.

SBP measured after the administered NE dose was defined as a reactive hypertensive episode if it was >120% than the baseline value. Bradycardia was defined as a heart rate < 50 bpm and was treated with an intravenous bolus of 0.5 mg atropine. In pregnant women who received a norepinephrine bolus before delivery, the two groups were compared in terms of neonatal outcomes. This included the APGAR scores for the newborn at 1st and 5th min post-delivery, as well as fetal umbilical arterial blood gas levels.

### 2.6. Primary and Secondary Outcomes

The primary outcome of this study was to determine the success rate of treatment using a 6 or 8 µg NE bolus in cases of maternal hypotension. Secondary outcomes included comparing (i) fetal umbilical blood gas values and (ii) APGAR scores in neonates born to mothers who received a NE bolus before delivery, (iii) determining the incidence of NE-induced reactive bradycardia and (iv) hypertension, and (v) comparing the groups for intraoperative nausea and vomiting.

### 2.7. Statistical Analysis and Sample Size Calculation

In a study, it was found that the success rate of treating spinal anesthesia-induced maternal hypotension with a 6 µg NE bolus dose was 77% [13]. Sample size calculation was performed using the clincalc.com/stats program. To achieve a 90% success rate, an absolute improvement of 17% in the management of a hypotensive episode was planned for sample size calculation. It was estimated that at least 134 mothers (67 per group) would be needed to have a study power of 80% and an alpha error of 0.05. To account for potential dropouts, this number was increased to 150 mothers (75 per group).

The patients’ data was analyzed using the Statistical Package for the Social Sciences (SPSS) for Windows version 25.0 (SPSS Inc., Chicago, IL, USA) program. During the analysis, normally distributed data were calculated as mean and standard deviation. For data that did not follow a normal distribution, median and quartile values were provided, as well as minimum and maximum values. Student’s *t*-test and Mann–Whitney U test were utilized to compare means of two independent groups, while the chi-square test was used to assess the relationship between categorical variables. A paired *t*-test was employed to compare two repeated continuous measurement means, and repeated ANOVA test was used to compare more than two repeated continuous measurement means. The statistical significance level was set at *p* < 0.05.

## 3. Results

**Figure 1:**
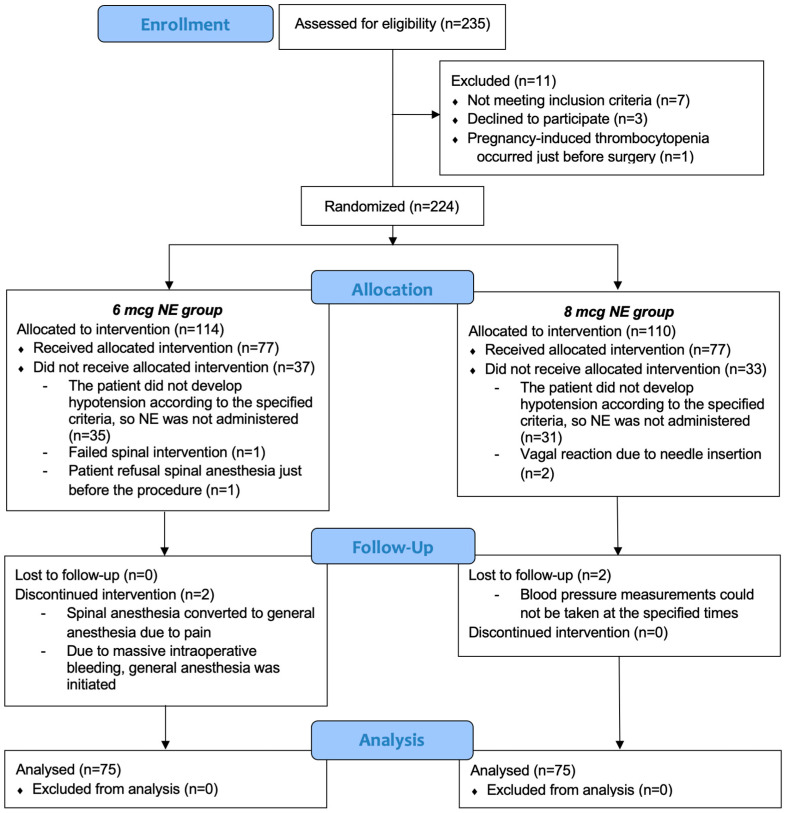
CONSORT flow diagram.

235 patients were screened for eligibility. Of these, 224 patients were randomized to receive either a bolus of 6 µg of NE or 8 µg of NE for the treatment of maternal hypotension induced by spinal anesthesia. 66 patients were excluded because no hypotension was observed during surgery after spinal anesthesia according to the determined criteria. Additionally, 8 patients were excluded from the final analysis for other reasons (Figure 1). Ultimately, 150 patients completed the study and were included in the final analysis (Group 6 µg, n = 75 vs. Group 8 µg, n = 75).

The demographic characteristics of the patients, preoperative assessments, and baseline hemodynamic values are summarized in Table 1. There were no significant differences in preoperative characteristics between the groups. While there was no difference between the groups in terms of total NE dose, the total number of hypotensive episodes was lower in the 8 µg group (245 vs. 178, *p* = 0.012). Furthermore, the success rate in treating hypotension was significantly higher in the 8 µg group (61.2% vs. 78.5%, *p* < 0.001). The frequency of reactive bradycardia requiring atropine after NE bolus was observed more frequently in the 6 µg group (Table 2).

In the study, maternal and neonatal outcomes were evaluated (Table 3). Nausea was more frequently observed in the 6 µg group (49.3% vs. 32%, *p* = 0.046), while there was no difference in vomiting. Neonatal outcomes were evaluated only in infants born to pregnant women who received NE during the pre-delivery period for hypotension. No difference was found between the groups in 1st and 5th min APGAR scores (*p* > 0.05). The groups were comparable in terms of umbilical artery blood gas pH values. In neonatal umbilical artery blood gas results, PCO_2_ was higher in the 8 µg group. The 8 µg group had better results for other umbilical blood gas parameters (PO_2_, HCO_3_ and base deficit) (Table 3).

**Figure 2:**
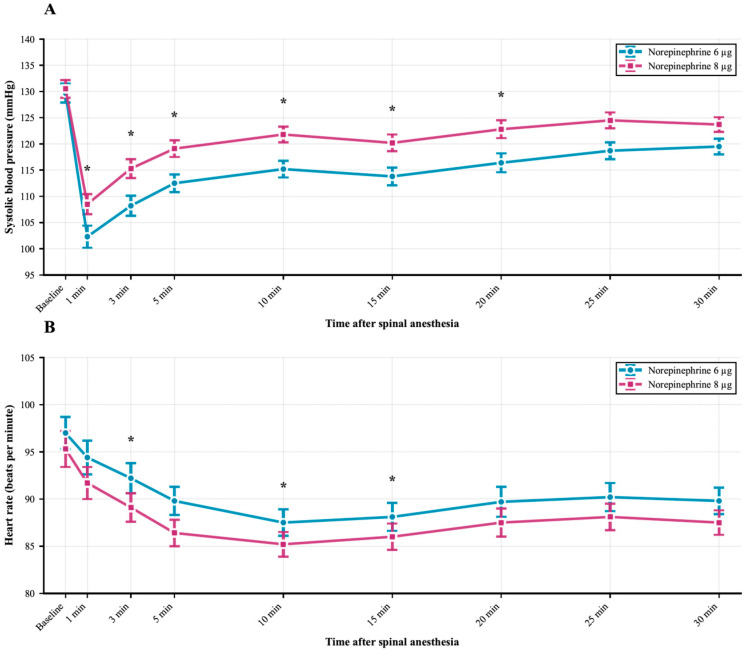
Time-course analysis of hemodynamic parameters following spinal anesthesia administration. (**A**) The 8 µg norepinephrine group maintained higher systolic blood pressure values compared to the 6 µg group, particularly evident from 1 min through 20 min after spinal anesthesia. (**B**) Heart rate changes over the perioperative period showed less pronounced but statistically significant differences at 3, 10, and 115 min intervals. Statistical significance was determined using repeated measures ANOVA with Bonferroni post hoc correction for multiple comparisons. Asterisks (*) denote statistically significant differences between groups at specific time points (*p* < 0.05).

**Figure 3:**
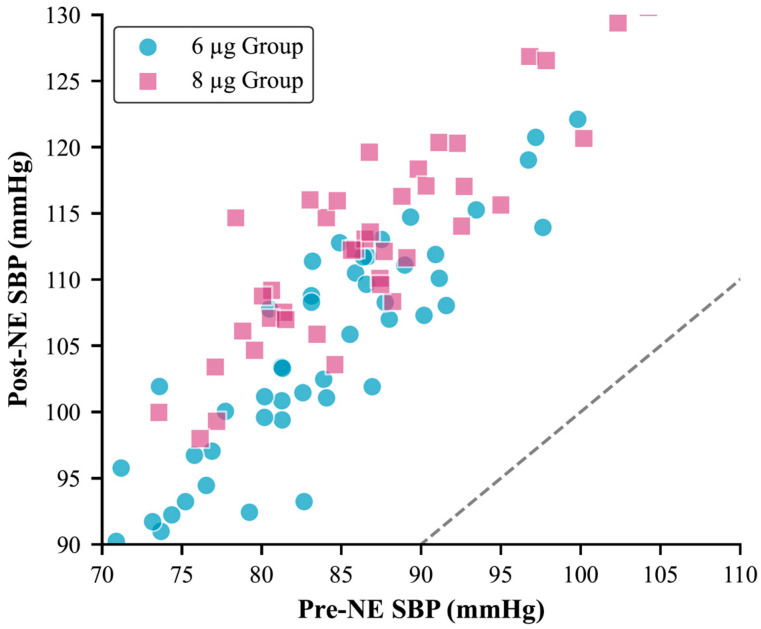
Scatter plot analysis of individual patient blood pressure responses to norepinephrine administration. The diagonal dashed line represents the line of equality (no change). Data points above this line indicate blood pressure improvement. Pre-treatment and post-treatment systolic blood pressure values demonstrate the superior therapeutic response achieved with the 8 µg dose regimen. Statistical analysis performed using linear regression analysis (*R*^2^ = 0.78 for 8 µg NE group vs. *R*^2^ = 0.65 for 6 µg NE group).

When comparing the SBP measurements obtained during surgery between groups, the 8 µg group showed significantly higher values from the 1st min after spinal block throughout the first 20 min compared to the 6 µg group (Figure 2A). Regarding HR, the measurements at the 3rd, 10th, and 15th min were significantly lower in the 8 µg group (Figure 2B). Between the two groups, the difference in SBP measured 1 min after NE administration and before NE administration was found to be significantly higher in the 8 µg group compared to the 6 µg group (Figure 3).

## 4. Discussion

In our study, we evaluated the response to a 6 or 8 µg norepinephrine bolus in the treatment of hypotension in patients undergoing cesarean section under spinal anesthesia. The 8 µg group showed fewer hypotensive episodes and required fewer norepinephrine boluses. Additionally, the success rate of hypotension treatment was higher in 8 µg group (61.2% vs. 78.5%, *p* < 0.001). The total norepinephrine dose administered was comparable between the groups. There was no difference in APGAR scores between the groups of neonates born to women who received norepinephrine before delivery. However, in the group with 8 µg NE, slightly better results were obtained in blood gas samples of the umbilical artery (PO_2_, HCO_3_ and base deficit), excluding the pH value.

Maintaining stable hemodynamic parameters during a cesarean section is crucial for achieving optimal maternal and neonatal outcomes [16]. Spinal anesthesia is preferred in most cases, and because the incidence of post-spinal hypotension is high, vasopressors have become important in the management of patients during a cesarean section. The International Consensus Statement on the management of hypotension with vasopressors during cesarean section recommends phenylephrine as the first-line treatment due to the sufficient supporting evidence of its advantages [17]. This consensus statement also suggests that vasopressors with α-1 agonism and moderate β-agonist activity may have the best profile. Additionally, heart rate is an important vital sign for this patient group, both for maintaining maternal cardiac output and placental blood flow [17,18]. Norepinephrine is an agent with strong α-1agonist and moderate β-agonist effects. Theoretically, norepinephrine is not expected to lower heart rate as much as phenylephrine because it contains a β-agonist effect. In a meta-analysis, NE was shown to be the vasopressor least likely to cause maternal bradycardia and superior to phenylephrine [19]. Consensus recommendations may have inherently accounted for the lack of sufficiently diverse and robust evidence for norepinephrine compared to phenylephrine [17,20].

An important prerequisite for accurately comparing the efficacy of two different vasopressors is calculating their equivalent doses when used for the same purpose. Most studies have found that phenylephrine and norepinephrine are similarly effective for preventing and treating post-spinal hypotension [6,21]. In a dose-finding study to prevent hypotension during cesarean delivery under spinal anesthesia, the required effective dose (ED90) for NE was 5.8 µg (95% CI, 5.01–6.59 µg, using isotonic regression method) in 90% of patients [14]. It is important to note that this study focused on prevention, not treatment of hypotension. Another study found an ED95 of NE to be 3.7 µg [22]. A study determining the ED50 of NE reported the required dose as 10 µg [23]. In many clinics, the typical phenylephrine bolus dose for this purpose has been reported as 100 µg [24]. Studies have reported relative potency ratios between phenylephrine and NE as 13.1:1 (100 µg phenylephrine equivalent to 7.7 µg NE), 11.3:1 (100 µg phenylephrine equivalent to 8.8 µg NE), and 17:1 (100 µg phenylephrine equivalent to 5.8 µg NE) [23]. The goal of these important studies was to establish the effective dose of NE to prevent hypotension and accurately determine its relative potency compared to phenylephrine, which is specified as the first-line drug recommended in guidelines. Studies in the literature compare boluses of 4 to 6 µg of NE and bolus doses [25] of 6 to 10 µg administered in addition to infusion [13]. However, there is a lack of data regarding NE dose comparisons for successfully treating hypotensive episodes. Therefore, the determination of NE doses in our study was based on this information. Upon reviewing the literature, no study comparing these doses clinically has been found.

When reviewing studies comparing the effectiveness of different NE bolus doses for treating maternal hypotension during CS, there is significant heterogeneity in methodology and study endpoints. In the study by Sarah et al., 5 µg and 10 µg NE bolus doses were compared for treating maternal hypotension and no difference was found between them [26]. However, that study included cases with severe hypotension, defined as systolic blood pressure falling below 60% of baseline. In another sequential dose-finding study, the prophylactic NE bolus dose was found to be 11 µg and the rescue dose was 12 µg [27]. Unlike our study, that study administered only combined spinal-epidural anesthesia to the patients, which could potentially alter the vasopressor requirements. In a study by Mohta et al., the effective NE bolus dose (ED95) was determined to be 7.2 µg [15]. That study also highlighted another important point: the success rate of a 7 µg NE bolus in treating spinal hypotension was 68% (n = 25), while at 8 µg, the success rate was 100% (n = 25). This finding serves as a reminder that small differences in NE bolus doses can lead to clinically meaningful differences in outcomes.

One study found that norepinephrine infusion was associated with higher cardiac output and heart rate and a lower incidence of bradycardia compared to phenylephrine infusion [28]. That study assessing the effects of cardiac output using thoracic bioreactance instead of systolic blood pressure found that norepinephrine provided higher cardiac index values than phenylephrine [28]. In a study by Hassabelnaby et al., heart rates were slightly lower in patients receiving a 10 µg bolus of NE compared to those receiving a 6 µg bolus of NE, but none required atropine [13]. Wang et al. compared the effects of 8 μg of norepinephrine or 100 μg of phenylephrine immediately after spinal anesthesia to prevent hypotension and found a lower incidence of bradycardia and higher cardiac output in patients receiving norepinephrine [29]. In our study, consistent with findings in the literature, mean heart rate values during surgery were lower in the 8 µg group than in the 6 µg NE group. However, the rate of reactive bradycardia was 8% in patients who received an 8 µg NE bolus, while it was 14.6% in the group that received a 6 µg bolus of NE.

Elective cesarean section patients who co-administered a simultaneous bolus of 4 or 6 µg norepinephrine with spinal anesthesia had nausea rates of 20% vs. 13.3%, bradycardia rates of 10% vs. 3.3%, respectively, and reactive hypertension was not detected in either group [26]. Prophylactic intravenous bolus dose of 6 μg norepinephrine when co-administered with spinal anesthesia was found to be more effective than 4 μg norepinephrine in terms of decreasing total rescue dose requirement of vasopressor and delaying the time to first rescue dose but without significant change in the incidence of hypotension and adverse effects in parturients undergoing CS under spinal anesthesia [25]. In the same study, the incidence of post spinal hypotension has been reported to be approximately 60–80%. In another study in which intermittent bolus NE (dose-finding study between 3 and 7 µg) was applied to patients with hypotension after spinal anesthesia, nausea was found in 27.5%, reactive bradycardia in 7.5%, and reactive hypertension in 10% [21]. In the same study, hypotension was defined as a drop below 100% of the baseline systolic blood pressure, thus the doses were applied almost prophylactically beyond treatment. In our study, nausea was less common in the 8 µg bolus NE group (49.3% vs. 32%), but there was no difference in vomiting (10.7% vs. 9.3%). It is well known that there is a positive correlation between the frequency of hypotensive episodes and nausea-vomiting after a spinal block. In our study groups, the total number of hypotensive episodes and the frequency of nausea were found to be correlated. We thought that the lack of difference in vomiting rates may be due to the fact that this complication was observed in a small number of patients.

Concerns exist regarding the safety profile of norepinephrine for newborns, as with any vasopressor agent administered to the mother before delivery. In a meta-analysis, norepinephrine was reported to be a better vasopressor than phenylephrine in terms of base deficit and fetal acid–base status on umbilical cord blood gas analysis [19]. Another meta-analysis was conducted to compare the effects of using phenylephrine or norepinephrine on the pH and base excess (BE) of the umbilical artery and vein in parturients undergoing cesarean section [6,7]. The analysis found no significant difference between phenylephrine and norepinephrine in terms of umbilical pH and base excess [6,7]. Subgroup analysis and meta-regression also failed to show any differences between the two agents. In our study, we found no difference between the groups in terms of umbilical blood gas pH. However, the group that received an 8 µg bolus of NE showed higher PCO_2_ values and better results in other umbilical blood gas parameters such as base deficit, PO_2_, and HCO_3_. However, these differences were small and of unclear clinical significance. This could be due to the lower number of hypotensive episodes in the 8 µg NE bolus group. Additionally, the group that received an 8 µg bolus of NE showed a greater increase in SBP and had a higher success rate in treating hypotension compared to the group that received a 6 µg bolus of NE. Theoretically, analysis of blood samples taken from umbilical vessels suggests that the placenta can partially break down catecholamines. Additionally, the use of norepinephrine may help reduce fetal stress immediately after birth by lowering fetal catecholamine levels.

Other concerns regarding norepinephrine use relate to the preferred venous route and its administration via bolus or infusion. A systematic review of the safety of vasopressor drug administration via peripheral venous cannula concluded that extravasation is rare and unlikely to lead to significant complications [30]. Extravasation has been reported more frequently in the use of small veins in the distal extremity, occurring in approximately 5% of cases [21]. In our study, the antecubital region was the preferred site, an 18–20 G cannula was inserted, and no complications were observed during the perioperative period. Some manufacturers do not mention the necessity of a central venous route for norepinephrine use, but they recommend caution due to the potential for tissue ischemia. Vasopressor drugs are often used in bolus form in addition to infusion, especially in the operating room setting. The International Consensus Statement recommends the use of variable-rate infusion devices [17]. However, in situations with a high number of patients, limited resources, and unexpected medical conditions, bolus administration provides a quick and effective approach.

Our study has some important limitations. We focused on SBP as the target for the primary outcome; however, the issue that led to the questioning of phenylephrine use in the first place relates to cardiac output. Although heart rate correlates well with cardiac output in this setting, the use of cardiac output monitoring to compare the efficacies of phenylephrine with norepinephrine would be highly useful and potentially more informative. The number of severe hypotensive episodes was not enough to compare the two study doses of NE. Our findings cannot be generalized to patients who undergo emergency cesarean sections or vaginal deliveries with bleeding, as well as those with serious comorbidities such as preeclampsia.

## 5. Conclusions

In conclusion, the success rate of treating maternal hypotension induced by spinal anesthesia, our primary outcome, was higher in the 8 µg NE bolus group. Additionally, although there was no difference in total NE consumption between the groups, we observed fewer hypotensive episodes, a lower number of norepinephrine bolus doses required, and less nausea in the 8 µg NE bolus group. Among neonatal outcomes, there was no difference in APGAR scores, but the 8 µg NE group showed better results in umbilical artery blood gas samples (PO_2_, HCO_3_, and base deficit), excluding the pH value. However, these differences in neonatal outcomes were small and of unclear clinical significance. Therefore, we have demonstrated that 8 µg NE bolus administration was more effective than that of 6 µg NE bolus in this clinical setting, without an increase in adverse effect rates.

## Figures and Tables

**Table 1:** Baseline demographic and operative characteristics of patients.

	Group 6 µg (n = 75)	Group 8 µg (n = 75)	*p* Value
Age (years)	31.43 (6.13)	31.31 (6.29)	0.906
Weight (kg)	75.99 (10.23)	78.12 (12.16)	0.357
Height (cm)	163.29 (5.85)	162.65 (6.09)	0.513
BMI (kg m^−2^)	28.5 (3.6)	29.6 (4.7)	0.119
ASA score, n (%)			
I	38 (50.7)	26 (34.7)	0.13
II	37 (49.3)	49 (65.3)	
Surgery type, n (%)			
Elective	52 (69.3)	57 (76)	0.464
Urgent for non-bleeding reasons	23 (30.7)	18 (24)	
Number of needle attempts for spinal block, n (%)			
1	50 (66.6)	55 (73.3)	0.822
2	18 (24.0)	16 (21.3)	
3	7 (9.4)	4 (5.4)	
Time from spinal block to delivery (min)	8.25 (2.34)	8.56 (2.87)	0.474
Surgery duration (min)	46.3 (8.3)	44.8 (9.7)	0.180
Baseline systolic blood pressure (mmHg)	129.73 (15.23)	130.48 (14.89)	0.762
Baseline diastolic blood pressure (mmHg)	80.7 (10.6)	80.1 (12.9)	0.741
Baseline heart rate (bpm)	97.00 (14.43)	95.31 (16.21)	0.267

Data presented as mean (standard deviation) or frequency (%). ASA: American Society of Anesthesiologists, BMI: body mass index.

**Table 2:** Characteristics of norepinephrine administration and treatment analysis of the hypotensive episodes.

Characteristics	Group 6 µg (n = 75)	Group 8 µg (n = 75)	*p* Value
*Pre-delivery period*			
Number of bolus NE	1 [1.0–2.0]	1 [1.0–2.0]	0.618
Amount of NE (µg)	6 [6.0–12.0]	8 [8.0–16.0]	**0.023**
Hypotensive episodes	60 (80)	57 (76)	0.562
Successful treatment rate (%)	77.5	81.4	0.562
*Total surgery period*			
Total number of bolus NE	3 [2.0–5.0]	2 [1.0–3.5]	**<0.001**
Total amount of NE (µg)	18 [12.0–30.0]	16 [8.0–28.0]	0.275
Total hypotensive episodes	245	178	**0.012**
Total successful treatment rate (%)	61.2	78.5	**<0.001**
Severe hypotensive episodes	2 (2.6)	0 (0)	0.497
Successfully treated severe episodes (%)	100.0	0.0	0.497
Increase rate of mean SBP after NE bolus, % (±SD)	20.5 (24.0)	25.4 (21.6)	**0.043**
Reactive hypertension after NE bolus	5 (6.7)	6 (8.0)	1.000
Reactive bradycardia after NE bolus	11(14.6)	6 (8.0)	**0.046**

Data presented as median [quartiles], frequency (%) or %(±SD). NE: norepinephrine, SBP: systolic blood pressure.

**Table 3:** Maternal and neonatal outcomes.

	Group 6 µg (n = 75)	Group 8 µg (n = 75)	*p* Value
*Maternal outcomes*			
Number of hypotensive episodes per mother	3.26 (2.68)	2.37 (2.18)	**<0.001**
Any complications	37 (49.3)	29 (38.7)	0.250
Incidence of nausea	37 (49.3)	24 (32.0)	**0.046**
Incidence of vomiting	8 (10.7)	7 (9.3)	0.578
Occurrence of high spinal block (above T4)	1 (1.3)	0 (0.0)	N/A
Headache	1 (1.3)	1 (1.3)	1.00
Dyspnea/chest pain	0 (0.0)	1 (1.3)	N/A
*Neonatal outcomes*			
Apgar score at 1st min	8 [7.5–9]	8 [8–9]	0.677
Apgar score at 5th min	9 [9–10]	10 [9–10]	0.416
Umbilical artery pH	7.32 [7.28–7.36]	7.33 [7.29–7.37]	0.161
Umbilical artery PO_2_ (mmHg)	15.70 [10.40–21.10]	22.50 [16.50–27.95]	**<0.001**
Umbilical artery PCO_2_ (mmHg)	42.80 [38.80–47.0]	46.00 [41.50–49.65]	**0.011**
Umbilical artery HCO_3_ (mmol L^−1^)	19.90 [18.70–21.70]	22.00 [19.60–23.85]	**<0.001**
Umbilical artery base excess (mmol L^−1^)	−1.80 [−3.35–1.55]	0.20 [−2.20–2.80]	**0.004**

## Data Availability

Data is available by contacting the corresponding author upon reasonable request.

## Abbreviations

The following abbreviations are used in this manuscript:

APGAR	Acronym. Appearance (skin color), Pulse (heart rate), Grimace (reflex irritability), Activity (muscle tone), and Respiration.
ASA	American Society of Anesthesiologists
CS	Cesarean section
HR	Heart rate
NE	Norepinephrine
SBP	Systolic blood pressure

## References

[1] Harrop S., Armstrong C.E. (2025). Spinal-induced hypotension at caesarean section. Anaesth. Intensiv. Care Med..

[2] Biava A.M., Cipriani G., Malja E., Bilotta F. (2025). Vasopressors for hypotension in spinal anesthesia for cesarean section. J. Anesth..

[3] Barud A.A., Ali I.A., Hilowle N.M., Hassan H.B., Osman I.M. (2025). Incidence and risk factors of hypotension in cesarean section patients under spinal anesthesia at a referral hospital in Mogadishu, Somalia. BMC Pregnancy Childbirth.

[4] Bhat A.D., Singh P.M., Palanisamy A. (2024). Neuraxial anaesthesia-induced hypotension during Caesarean section. BJA Educ..

[5] Sonsale A.R., Kuttarmare S.M. (2025). Comparison of phenylephrine versus ephedrine in managing maternal hypotension during cesarean section under spinal anesthesia. Eur. J. Cardiovasc. Med..

[6] Zhao S., Chen Q., Qin P., Liu L., Wei K. (2024). Comparison of vasopressors for management of hypotension in high-risk caesarean section under neuraxial anesthesia: A systematic review and network meta-analysis. BMC Anesthesiol..

[7] Kang H., Sung T.Y., Jee Y.S., Kwon W., Cho S.-A., Ahn S., Cho C.-K. (2024). A Comparison of Norepinephrine versus Phenylephrine to Prevent Hypotension after Spinal Anesthesia for Cesarean Section: Systematic Review and Meta-Analysis. J. Pers. Med..

[8] Singh A., Jain K., Goel N., Arora A., Kumar P. (2022). Neonatal outcomes following prophylactic administration of phenylephrine or noradrenaline in women undergoing scheduled caesarean delivery: A randomised clinical trial. Eur. J. Anaesthesiol..

[9] Guo L., Qin R., Ren X., Han C., Xue W., He L., Ma L., Pan H., Ma S., Chen Y. (2022). Prophylactic norepinephrine or phenylephrine infusion for bradycardia and post-spinal anaesthesia hypotension in patients with preeclampsia during Caesarean delivery: A randomised controlled trial. Br. J. Anaesth..

[10] Chen Z., Zhou J., Wan L., Huang H. (2022). Norepinephrine versus phenylephrine infusion for preventing postspinal hypotension during cesarean section for twin pregnancy: A double-blinded randomized controlled clinical trial. BMC Anesthesiol..

[11] Du W., Song Y., Li J., Zhou X., Xu Z., Liu Z. (2022). Comparison of Prophylactic Norepinephrine and Phenylephrine Infusions During Spinal Anaesthesia for Primary Caesarean Delivery in Twin Pregnancies: A Randomized Double-Blinded Clinical Study. Drug Des. Devel. Ther..

[12] Zhou Y., Yu Y., Chu M., Zhang Y., Yu X., Chen G. (2022). Comparison of Metaraminol, Phenylephrine, and Norepinephrine Infusion for Prevention of Hypotension During Combined Spinal-Epidural Anaesthesia for Elective Caesarean Section: A Three-Arm, Randomized, Double-Blind, Non-Inferiority Trial. Drug Des. Devel. Ther..

[13] Hassabelnaby Y.S., Hasanin A.M., Adly N., Mostafa M.M.A., Refaat S., Fouad E., Elsonbaty M., Hussein H.A., Mahmoud M., Abdelwahab Y.M. (2020). Comparison of two Norepinephrine rescue bolus for Management of Post-spinal Hypotension during Cesarean Delivery: A randomized controlled trial. BMC Anesthesiol..

[14] Ngan Kee W.D., Lee S.W., Ng F.F., Tan P.E., Khaw K.S. (2015). Randomized double-blinded comparison of norepinephrine and phenylephrine for maintenance of blood pressure during spinal anesthesia for cesarean delivery. Anesthesiology.

[15] Mohta M., Kumar R., Salhotra R., Kumari S., Mulodhia P. (2025). Relative potency of norepinephrine and mephentermine bolus for the treatment of spinal hypotension during elective caesarean delivery: A randomized, blinded up-down sequential allocation study. Int. J. Obstet. Anesth..

[16] Mohta M. (2023). Norepinephrine—Can it Replace Phenylephrine as the Vasopressor of Choice in Obstetric Anesthesia?. J. Indian Coll. Anaesthesiol..

[17] Kinsella S.M., Carvalho B., Dyer R.A., Fernando R., McDonnell N., Mercier F.J., Palanisamy A., Sia A.T.H., Van de Velde M., Vercueil A. (2018). International consensus statement on the management of hypotension with vasopressors during caesarean section under spinal anaesthesia. Anaesthesia.

[18] Mets B. (2016). Should Norepinephrine, Rather than Phenylephrine, Be Considered the Primary Vasopressor in Anesthetic Practice?. Anesth. Analg..

[19] Singh P.M., Singh N.P., Reschke M., Ngan Kee W.D., Palanisamy A., Monks D.T. (2020). Vasopressor drugs for the prevention and treatment of hypotension during neuraxial anaesthesia for Caesarean delivery: A Bayesian network meta-analysis of fetal and maternal outcomes. Br. J. Anaesth..

[20] Herbosa G.A.B., Tho N.N., Gapay A.A., Lorsomradee S., Thang C.Q. (2022). Consensus on the Southeast Asian management of hypotension using vasopressors and adjunct modalities during cesarean section under spinal anesthesia. J. Anesth. Analg. Crit. Care.

[21] Onwochei D.N., Ngan Kee W.D., Fung L., Downey K., Ye X.Y., Carvalho J.C.A. (2017). Norepinephrine Intermittent Intravenous Boluses to Prevent Hypotension During Spinal Anesthesia for Cesarean Delivery: A Sequential Allocation Dose-Finding Study. Anesth. Analg..

[22] Mohta M., Dubey M., Malhotra R.K., Tyagi A. (2019). Comparison of the potency of phenylephrine and norepinephrine bolus doses used to treat post-spinal hypotension during elective caesarean section. Int. J. Obstet. Anesth..

[23] Ngan Kee W.D. (2017). A random-allocation graded dose–response study of norepinephrine and phenylephrine for treating hypotension during spinal anesthesia for cesarean delivery. Anesthesiology.

[24] George R.B., McKeen D.M., Dominguez J.E., Allen T.K., Doyle P.A., Habib A.S. (2018). A randomized trial of phenylephrine infusion versus bolus dosing for nausea and vomiting during cesarean delivery in obese women. Can. J. Anaesth..

[25] Choudhary S., Dagar R., Jeenger L., Bhiwal A.K., Tuteja S., Gupta S. (2020). Prophylactic co-administration of two different bolus doses of norepinephrine in spinal-induced hypotension during caesarean section: A prospective randomized double-blinded study. J. Obstet. Anaesth. Crit. Care.

[26] Amin S.M., Hasanin A., Ghanem N.T., Mostafa M., Elzayat N., Elsherbiny M., Abdelwahab Y. (2024). Comparison of Two Norepinephrine Rescue Bolus Doses for Management of Severe Post-Spinal Hypotension During Elective Caesarean Delivery: A Randomized, Controlled Trial. Int. J. Gen. Med..

[27] Xu T., Zheng J., An X.H., Xu Z.F., Wang F. (2019). Norepinephrine intravenous prophylactic bolus versus rescue bolus to prevent and treat maternal hypotension after combined spinal and epidural anesthesia during cesarean delivery: A sequential dose-finding study. Ann. Transl. Med..

[28] Belin O., Casteres C., Alouini S., Le Pape M., Dupont A., Boulain T. (2023). Manually Controlled, Continuous Infusion of Phenylephrine or Norepinephrine for Maintenance of Blood Pressure and Cardiac Output During Spinal Anesthesia for Cesarean Delivery: A Double-Blinded Randomized Study. Anesth. Analg..

[29] Wang X., Mao M., Zhang S.S., Wang Z.H., Xu S.Q., Shen X.F. (2020). Bolus norepinephrine and phenylephrine for maternal hypotension during elective cesarean section with spinal anesthesia: A randomized, double-blinded study. Chin. Med. J..

[30] Tian D.H., Smyth C., Keijzers G., Macdonald S.P., Peake S., Udy A., Delaney A. (2020). Safety of peripheral administration of vasopressor medications: A systematic review. Emerg. Med. Australas..

